# Health risk assessment of organochlorine pesticide residues in edible tissue of seafood

**DOI:** 10.3389/fvets.2022.1042956

**Published:** 2022-11-28

**Authors:** Mohamed A. Hussein, Omnya S. Hammad, Ahmed E. Tharwat, Wageh S. Darwish, Ahmed Sayed-Ahmed, František Zigo, Zuzana Farkašová, Ibrahim F. Rehan

**Affiliations:** ^1^Department of Food Control, Faculty of Veterinary Medicine, Zagazig University, Zagazig, Egypt; ^2^Department of Anatomy and Embryology, Faculty of Veterinary Medicine, Menoufia University, Shebin Alkom, Egypt; ^3^Department of Nutrition and Animal Husbandry, University of Veterinary Medicine and Pharmacy, Košice, Slovakia; ^4^Department of Husbandry and Development of Animal Wealth, Faculty of Veterinary Medicine, Menoufia University, Shebin Alkom, Egypt; ^5^Department of Pathobiochemistry, Faculty of Pharmacy, Meijo University Yagotoyama, Nagoya-shi, Japan

**Keywords:** target hazard quotient, seafood, health risk assessment, hazard index, organochlorine pesticides (OCPs)

## Abstract

Fish is one of the most valuable foods with high-quality animal protein. However, aquaculture, or ingesting contaminated food, allows organochlorine pesticides (OCPs) to enter the fish's body, and therefore, it negatively impacted public health. One-hundred and twenty random samples of *Clupea harengus* (*C. harengus*)*, Mugil cephalus* (*M. cephalus*)*, Sardinella aurita* (*S. aurita*)*, Oreochromis niloticus* (*O. niloticus*)*, Neptunus pelagicus* (*N. pelagicus*) and *Sepia savigngi* (*S. savigngi*) (*n* = 20 each) were collected from local markets in Mansoura city, Egypt. Samples were checked to see whether any residues of OCPs with the application of risk assessment due to their consumption by Mansoura citizens. The findings indicated that summation hexachlorocyclohexane (∑HCH) in examined seafood samples ranged from 0.27 ± 0.13 in *N. pelagicus* to 61.61 ± 52.03 μg.kg^−1^ in *S. aurita*. Also, the γ-HCH isomer was considered the more prominent among isomers. Hexachlorobenzene (HCB) was found in five different species, with mean values of 2.03 ± 1.85, 1.5.7 ± 1.17, 0.94 ± 0.87, 0.35 ± 0.06, and 0.18 ± 0.06 μg.kg^−1^ in *C. harengus, S. aurita, M. cephlaus, O. niloticus*, and *S. savigngi*. Moreover, summation of Heptachlors (∑HPTs) was 10.19 ± 7.63, 1.27 ± 0.26, 2.58 ± 0.11, 0.95 ± 0.12, 0.21 ± 0.11 and 0.32 ± 0.03 μg.kg^−1^ of wet weight in examined *C. harengus, M. cephlaus, S. aurita, O. niloticus, N. pelagicus*, and *S. savigngi*. Aldrin and dieldrin residues were 3.75 ± 1.31 and 4.86 ± 1.33 μg.kg^−1^ in *C. harengu*, meanwhile they were 1.61 ± 0.77 and 0.78 ± 0.04 μg.kg^−1^in *M. cephalus*. Dichlorodiphenyldichloroethylene (pp-DDE) was dominant in all examined species within different concentrations 5.08 ± 4.12, 0.98 ± 0.10, 3.07 ± 0.91, 0.93 ± 0.27, 0.08 ± 0.01 and 0.35 ± 0.02 μg.kg^−1^ in *C. harengus, M. cephlaus, S. aurita, O. niloticus, N. pelagicus* and *S. savigngi*, respectively. We concluded that all examined seafood samples were lower than the recommended maximum residue limit. Also, the estimated daily intake was less than the permitted daily intake. Non-carcinogenic indices of target hazard quotient and hazard index for OCPs in all examined species were less than 1.

## Graphical Abstract

Schematic cartoon of the experimental protocol – Created on BioRender.com. 
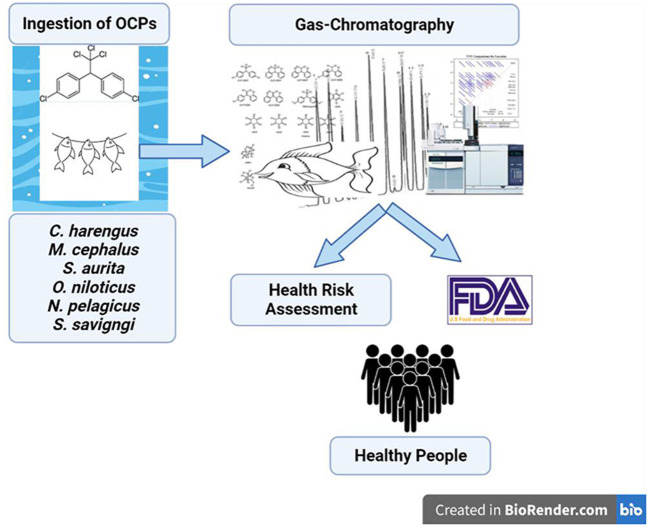

## Introduction

Fish is considered one of the most valuable foods because it provides Egyptian consumers with high biological value animal protein while also addressing the issue of animal meat scarcity (1). The total fish production in Egypt was 2.2 MMT (million metric tons) in 2021 with aquaculture share at 1.7 MMT of total production. Egypt has a promising plan to raise fish production to 3 MMT by 2025 (2). Moreover, fish consumption is strongly advised for children's nutrition, and therefore, the safety and quality of the product should always be checked to safeguard public health (3). The unpredictable and flighty utilization of pesticides in horticulture creates ecological issues; particularly for the aquatic environment by changing the nature of water and influencing the biochemical and physiological merits of non-target fish (4). Different routes, such as direct contact with water or ingesting contaminated food, allow organochlorine pesticides (OCPs) to enter the fish's body (5). The pollution load in fish is affected by the level of pollution in the water. As a result, fish are the most important bioindicators of pesticide contamination in water bodies (6). OCPs, that remains in the environment, bioaccumulate in organisms, and are hazardous to both humans and wildlife, becoming a major worry for the world in recent years. OCP bioaccumulation in the biota can have negative consequences for immunity, the neurological system, and reproduction (7). The OCPs have lipophilic nature, hydrophobicity, and low chemical and biological degradation rates that prompt their bioaccumulation and subsequent magnification in beings of the food chain (8). The primary cause of pesticide pollution is food contaminated with pesticides. It was assumed that because fish is the final animal in the food chain, it is responsible for more than 80% of all pesticide residues consumed by humans. It is the one most affected by it, and society as a whole has subsequently grown more worried about the potential damage to human health posed by eating such contaminated biota (9). Furthermore, several recent studies in Japan and China have observed a correlation between eating fish and serum organochlorine levels in humans (10, 11). Since late 1990, the importation and use of OCPs have been outlawed in Egypt after more than 50 years of use in agriculture and public health. However, some OCPs, including DDT and -HCH (lindane), are still used illegally by farmers due to their low cost, simplicity of use, and ability to effectively eradicate pests (12, 13). Egypt reportedly utilized 45,000 MT of toxaphene (1955–1961), 10,500 MT of endrin (1961–1981), 13,500 MT of DDT (1952–1971), and 11,300 MT of lindane (1952–1978) over 30 years, switching between the various chemicals regularly (14). Therefore, the objective of this study was to assess the presence of OCPs and public health hazards associated with the consumption of *C. harengus, M. cephalus, S. aurita, O. niloticus, N. pelagicus, and S. savigngi* marketed in Mansoura city, Egypt.

## Materials and methods

### Collection and preparation of samples

One-hundred and twenty samples of *C. harengus, M. cephalus, S. aurita, O. niloticus, N. pelagicus, and S. savigngi* (*n* = 20 each) were collected randomly from local markets in Mansoura, Egypt. *C. harengus* is usually imported from Holland. However, *O. niloticus* and *M. cephalus* are usually farmed in Egypt, *S. pilchards* from the Mediterranean Sea, and *S. savigngi* and *N. pelagicus* from the Red Sea. The fish samples were collected from the Mansoura City fish market in Egypt (marketed fish for human consumption). We study the safety of fish for consumers – The fish sample collection site is not the same place for fishing so we are of not need a map. Those samples were maintained in an ice box and directly shipped to the Department of Food Control, Faculty of Veterinary Medicine, Zagazig University. To get rid of dirt and other entrapped materials before being distinguished and given remarkably recognizable proof codes, the samples were washed many times with deionized water. Using a stainless-steel knife, gut each fish sample to remove the intestines, scales, head, tails, fins, and bones. Remove the edible portion of the samples (50 g) for extraction and cleaning procedures.

###  Chemicals

Sigma-Aldrich Chemie GmbH provided standard OCPs such as pp-DDT, pp-DDD, pp-DDE, HCH, heptachlor, heptachlor epoxide, aldrin, endrin, chlordane, methoxychlor, and HCB (Kappelweg, Schnelldor, Germany). Other chemicals were purchased from Merck and were of the highest quality available (Darmstadt, Germany). Dichloromethane (DCM) was purchased from J.T. Baker (Phillipsburg, NJ, USA). Acetonitrile (ACN, GC-MS SupraSolv^®^) and *n*-hexane (HEX, Ultra Resi-Analyzed) were purchased from Merck KGaA (Darmstadt, Germany).

### Sample extraction, preparation, partitioning, and cleaning

In Egypt, the consumer eats the muscle of fish only. Also, the regulation and risk analysis adopted for muscle. Each muscle sample (50 g) was mixed with anhydrous sodium sulfate (100 g) and petroleum ether for 2 min for three successive extraction steps (15). Samples were dried by solvent evaporation in a rotary evaporator at 40°C after filtration. During the portioning of the extracted samples, according to the previous report (16). The extracted samples were separated using an 80:20 n-hexanes and acetonitrile solution in a volume of 100 mL. The acetonitrile layer was collected and evaporated on a rotary evaporator to a 10 mL volume for use in Florisil cleanup after three partitioning cycles. The Florisil was provided by Silica (Silica Co., USA). The solvents were all of pesticide quality. Florisil was activated at 130°C for 24 h and then cooled at 25°C. A chromatographic column containing 20 g of activated Florisil was used to clean the extracted samples. The eluent was cleaned up, evaporated on a rotary evaporator, and then dissolved in 10 mL of hexane. An aliquot of each extract was transferred to 2 mL injection vials in order to get it ready for the electron capture gas chromatography examination (17).

### Determination of OCPs concentrations

The maximum residue limit (MRL) of OCPs is proposed by the Food and Drug Administration (FDA) since the MRL has not been established by the WHO yet (12, 13). Organochlorine pesticide residues were evaluated using electron capture gas chromatography (Hewlett Packard GC Model 6890) equipped with a Ni_63_-electron capture detector. An HP- 5MS capillary column (30 m in length, 0.32 mm inner diameter, and 0.25 μm film thickness, with N_2_ as the carrier gas and a flow rate of 4 mL/min, respectively) was used in the gas chromatograph (GC). The extract was injected into a single input that was split into two columns. The instrumental settings were as follows: The temperature program for the gas chromatography oven began at 150°C for 5 min, increased to 170°C at a rate of 5 °C/min and held for 10 min, then increased to 220°C at a rate of 10°C/min and held for 20 min (with a total run time of 44 min); the injection volume was 1 ll and the flow rate of nitrogen gas was 20 mL/min. Analytical process quality control was carried out based on a comparison with the developed standard curves and injector and detector temperatures of 230°C. To verify the analytical procedures, the standard reference material, SRM 1947 (Lake Michigan Fish Tissue), was handled and inspected comparably to the samples. Recovery rates for the examined OCPs ranged from 88 to 106%.

### Health risk assessment

Estimated daily intake (EDI) was determined by factoring in data on organochlorine analyses, fish intake patterns, and adult Egyptian body weight. (μg/kg/day) was obtained following the subsequent equation: EDI = (Cm x FIR)/BW. Where, Cm = organochlorine concentration in fish (μg.kg^−1^), FIR = the food ingestion rate, estimated at 48.57g/day (18), and BW = the average body weight of an adult is 70 kg, then compared to acceptable daily intakes (ADIs).

The target hazard quotient (THQ) was employed to describe the health risk that the consumption of seafood poses to the Egyptian population. The exposure to reference dosages (RfD) ratio is shown below, refers to the amount of a contaminant that a person could be exposed to every day for the rest of their life without significantly increasing the probability that it would cause harm. Rfd was 0.0003, 0.0008, 0.0005, 0.00003, 0.0005, and 0.0005 (g/g bw/day), respectively, for HCH, HCB, HPTs, Drins, CHLs, and DDTs (19, 20). If the ratio is less than 1, the population would not be at risk; if it's equal to or higher than 1, the population will be at risk of health problems. It uses the equation shown below (21):


$$\begin{array}{l} THQ=\frac{EF\;\times \;ED\;\times \;FIR\;\times \;C}{RFD\;\times \;BW\;\times ÃT}\times 10^{-3} \end{array}$$

THQ stands for target hazard quotient, EF for exposure frequency (365 days annually), and ED for exposure duration (average lifetime of 70 years); FIR stands for food ingestion rate (g/day); C stands for organochlorine concentration in fish (g/g); RfD stands for oral reference dose (mg/kg/day); BW stands for average adult body weight (70 kg); and AT stands for the average exposure time (365 days /year, assuming a lifespan of 70 years).

The hazard index (HI) has been performed to assess the probable human health hazard of more than one organochlorine. The HI refers to the sum of all THQ for various organochlorine exposures as shown in the equation below: HI = ΣTTHQs = THQ HCH + THQ HCB + THQ HPTs +THQ Drins +THQ CHLs +THQ DDTs where Σ TTHQs is the target hazard quotients of all organochlorines; while, the hazard index becomes over 1, the possible human health risk is expected (22).

### Statistical analysis

A completely randomized design (CRD) was used to statistically evaluate the data with a significance level of *P* ≤ 0.05. SAS 9.3 was used for all statistical analyses (SAS Institute Inc., Cary, NC, USA). The information was displayed as means and relative standard deviation (RSD).

## Results and discussion

### Organochlorine pesticide residues

There are five main isomeric forms of hexachlorocyclohexane (HCH): α-HCH, β-HCH, γ-HCH, δ-HCH, and ε-HCH. The most common isomer of HCH is called α-HCH (23). Among these isomers, only the γ-HCH isomer, which is sold as Lindane, has insecticidal properties (24). The main component of several commercial medications used to treat scabies and head lice in children is lindane, which is manufactured as a shampoo or lotion (25). Also, the γ*-*HCH has been used to treat soil, animals, pets, forestry products, seeds, and seedlings, according to the previous report (26). Because HCHs are particularly poisonous to aquatic organisms, they are highly susceptible to bioaccumulation (27). The downward concentrations of ∑HCH in seafood samples were determined, *S. aurita* (61.61 ± 52.03 μg.kg ^1^) > *C. harengus* (19.7 ± 11.64 μg.kg^−1^) > *O. niloticus* (2.32 ± 0.83 μg.kg^−1^) > *M. cephlaus* (1.33 ± 0.74 μg.kg^−1^) > *S. savigngi* (1.43 ± 0.70 μg.kg^−1^) > *N. pelagicus* (0.27 ± 0.13 μg.kg^−1^) as shown in Table 1. The ∑HCH was detected previously in seafoods worldwide at concentrations 38.82 μg.kg^−1^ in Kenya (from twelve fish species; *Lethrinus “harak, ribulose”, Siganus rivulatus, Litianus russeli, Tilapia zilli, Mondaclilus argentus, Epinephelus Caeruleopantantus, Carcharhinus macloti, Valamugil buchsrhinus, Sardinella fimbrita, Penaeus sp., Apolectus niger, and Pampus argenteus*) (28). Moreover, ∑HCH was 62.95 μg.kg^−1^ in Cote d'Ivoire (from two species of fish, *Oreochromis niloticus*, and *Chrysichthys nigrodigitatus*) (29). Also, ∑HCH was 6910 μg.kg^−1^ in Nigeria (from three species of fish, *Tilapia zilli, Ethmalosa fimbriata*, and *Chrysichthys nigrodigitatus*) (30). Additionally, ∑HCH was 23.8 μg.kg^−1^ in Turkey (from eighteen fish species (trout, red mullet, bluefish, gilt-headbream, sea bass, horse mackerel, goby, gray mullet, gurnard, greater amberjack, common sea bream, whiting, bonito, pilchard, native mackerel, import mackerel, pike perch, and garfish) (31). In Africa, ∑HCH was 15.44 μg.kg^−1^ in Ghana (from six fish species, *Heterotis niloticus, Channa obscura, Hepsetus odoe, Tilapia zilli, Clarias gariepinus, and Chrysichthys nigrodigitatus*) (32), 1.1 μg.kg^−1^ in Ethiopia (from six fish species, *Oreochromis niloticus, Clarias gariepinus, Barbus intermedius, Barbus paludinosus, Garra quadrimaculata*, and *Aplocheilichthyes antinorii*) (17) and 0.88 μg.kg^−1^ in Egypt (from *Tilapia nilotica*) (33). Regarding HCH isomer concentration in this study, γ-HCH isomer was considered the more prominent between isomers. Nearly similar findings for α-HCH, β-HCH, γ-HCH and δ-HCH were 0.22, 0.17, 0.52, and 0.19 μg.kg^−1^ in Ethiopian fish (17). Also, β-HCH and γ-HCH were 5.5 and 25.38 μg.kg^−1^ in fish samples collected from Ghana (34). Regarding the obtained results, all examined samples were below the MRL 300 μg.kg^−1^ (35).

**Table 1:** Incidence and residual level (mean ± SD) of Hexachlorocyclohexane (HCH), Hexachlorobenzene (HCB), and Heptachlor (HPTs) in examined seafood species (μg.kg^−1^) of wet weight (*n* = 20 each).

		**α-HCH**	**β-HCH**	**γ-HCH**	**δ-HCH**	**∑HCH**	**HCB**	**Heptachlor**	***cis*** **-Hep-epox**	***trans*** **-Hep-epox**	**∑HPTs**
*C. harengus*	Incidence	95%	50%	100%	60%		100%	95%	25%	75%	
	Mean ± SD	3.21 ± 1.81	4.02 ± 3.07	7.82 ± 4.11	4.02 ± 2.65	19.7 ± 11.64^b^	2.03 ± 1.85	2.35 ± 1.58	5.61 ± 4.69	2.22 ± 1.36	10.19 ± 7.63^a^
*M. cephalus*	Incidence	100%	ND	ND	ND		60%	100%	50%	ND	
	Mean ± SD	1.33 ± 0.74	ND	ND	ND	1.33 ± 0.74^cd^	0.94 ± 0.87	0.63 ± 0.14	0.64 ± 0.12	ND	1.27 ± 0.26^b^
*S. aurita*	Incidence	70%	70%	85%	ND		70%	ND	ND	100%	
	Mean ± SD	4.13 ± 1.91	12.65 ± 14.65	44.84 ± 35.47	ND	61.61 ± 52.03^a^	1.57 ± 1.17	ND	ND	2.58 ± 0.11	2.58 ± 0.11^b^
*T. nilotica*	Incidence	100%	70%	50%	50%		85%	ND	70%	50%	
	Mean ± SD	0.75 ± 0.53	0.51 ± 0.08	0.53 ± 0.16	0.53 ± 0.05	2.32 ± 0.83^c^	0.35 ± 0.06	ND	0.45 ± 0.02	0.50 ± 0.10	0.95 ± 0.12^bc^
*N. pelagicus*	Incidence	ND	100%	ND	70%		ND	100%	ND	100%	
	Mean ± SD	ND	0.09 ± 0.01	ND	0.18 ± 0.12	0.27 ± 0.13^d^	ND	0.15 ± 0.1	ND	0.06 ± 0.01	0.21 ± 0.11^c^
*S. savigngi*	Incidence		ND	25%	50%		40%	ND	ND	25%	
	Mean ± SD	0.27 ± 0.13	ND	0.67 ± 0.49	0.49 ± 0.08	1.43 ± 0.70^cd^	0.18 ± 0.06	ND	ND	0.32 ± 0.03	0.32 ± 0.03^c^

a, b, c, d Columns that carry different superscript letters are significantly different at P < 0.05. SAS 9.3 was used for all statistical analyses (SAS Institute Inc., Cary, NC, USA).

Hexachlorobenzene (HCB), chlorinated benzene, has been applied to wheat seeds as a fungicide to prevent fungal infections. Since its introduction as a fungicide in 1945, HCB has been detected in all forms of food as a result of the accumulation of this substance in high-fat foods, such as dairy products, eggs, animal fats, and some fish (36). There has been a continuous decrease in the amount of HCB in the environment during the last 20–25 years. However, there is still a lot of this chemical in the environment (37). The HCB residues were detected in 100, 70, 60, 85, and 40% with mean values of 2.03 ± 1.85, 1.5.7 ± 1.17, 0.94 ± 0.87, 0.35 ± 0.06 and 0.18 ± 0.06 μg.kg^−1^ in examined *C. harengus, S. aurita, M. cephlaus, O. niloticus*, and *S. savigngi*, respectively. Meanwhile, it was not detected in *N. pelagicus* (Table 1). All examined samples were below the MRL 200 μg.kg^−1^ (38). *C. harengus* significantly contained higher HCB residues than other species (*P* < 0.05). The findings show that species-specific ecological traits, such as eating patterns and habitat, have a role in the bioaccumulation of OCPs in fish. HCB residues of 0.80–1.68 μg.kg^−1^ were in Spain (39) and 1.78 μg.kg^−1^ was detected in fish caught off the coast of South Africa (40). Meanwhile, higher HCB residues of 175.10 μg.kg^−1^ were detected in fish samples from Cote d'Ivoire (29), and 22.93 μg.kg^−1^ was detected in Nigerian fish samples (41). There have been reports of lower HCB levels in Italian and European markets (mean: 0.06; range: 0.03–0.15 g.kg^−1^ of wet weight) (42).

Heptachlor is a cyclodiene pesticide used to control termites and as an insecticide on food and seed crops (24). The primary metabolite of heptachlor, heptachlor epoxide, is quite persistent in soil. 14–16 years after treatment, traces of heptachlor epoxide have occasionally been detected in soil. Heptachlor epoxide can be directly absorbed by plants from the soil, and it bioaccumulates in mammals. In more than 60 nations, it has been outlawed or regulated (43). Three isomers heptachlor, *cis*-Heptachor-epoxide, and *trans*-Heptachlor-epoxide were detected only in *C. harengus* examined samples at concentrations 2.35 ± 1.58, 5.61 ± 4.69 and 2.22 ± 1.36 μg.kg^−1^of wet weight, respectively indicate the past and recent exposure to heptachlor. While *trans*-Heptachlor-epoxide *S. aurati* and *S. savigngi* at concentrations 2.58 ± 0.11 and 0.32 ± 0.03 μg.kg^−1^ of wet weight indicate the past exposure. The summation of heptachlor (∑HPTs) were 10.19 ± 7.63, 1.27 ± 0.26, 2.58 ± 0.11, 0.95 ± 0.12, 0.21 ± 0.11 and 0.32 ± 0.03 μg.kg^−1^ of wet weight in examined *C. harengus, M. cephlaus, S. aurita, O. niloticus, N. pelagicus*, and *S. savigngi*, respectively (Table 1). The difference between *C. harengus* and the other investigated species was significant (*P* < 0.05), which may be related to its ongoing exposure to heptachlor in the environment. All examined species were below the permissible limits for heptachlor in edible seafood at 300 μg.kg^−1^of wet weight (35). The herein ∑HPTs in this study were lower than 35.33 μg.kg^−1^ in fish collected from Cote d'Ivoire (29) and 40.18 μg.kg^−1^ in those from Nigeria (41). The lower ∑HPTs were reported in Ethiopia and Egypt at 0.57 and 0.65 μg.kg^−1^ (17, 33).

Aldrin and dieldrin are substances that have been extensively utilized in public health applications to stop mosquito-borne diseases including malaria and sleeping sickness as well as to control insects in agriculture (24). Aldrin changes into dieldrin in living systems, while dieldrin is resistant to bacterial and chemical destruction outside of living systems (32). Dieldrin's stereoisomer, endrin, exists. It tends to be stationary, adsorbs soil particles, and is hydrophobic. Endrin has largely been utilized as an avicide, rodenticide, and insecticide (44). The aldrin residues were detected in 20%, 85%, 50% and 100% with mean values of 3.75 ± 1.31, 1.61 ± 0.77, 0.88 ± 0.31 and 0.09 ± 0.01 μg.kg^−1^ in *C. harengus, M. cephalus, O. niloticus*, and *N. pelagicus*, respectively. Aldrin residues were found in Turkey, Nigeria, and Ghana at quantities ranging from 3.3 to 26.1, 18.47, and 1.02 μg.kg^−1^ (31, 34, 41). The dieldrin mean values were 4.86 ± 1.33, 0.78 ± 0.04 and 0.55 ± 0.02 μg.kg^−1^ in *C. harengus, M. cephalus*, and *O. niloticus*, respectively. On the contrary, Table 2 demonstrates that not all samples had endrin. The results can be explained by the previous report (24), which reported that endrin was extremely tenacious, but when exposed to sunshine, it partially breaks down into endrin ketone and endrin aldehyde. Higher dieldrin residues were obtained at 109.38 and 3,098 and 67.96 μg.kg^−1^ in Kenya and Nigeria (28, 45). Meanwhile, lower values 0.04 and 0.02 μg.kg^−1^ in Ghana and Ethiopia (46, 47). All obtained values were lower than the permissible according to the previous report (35), Aldrin, dieldrin, and endrin in fish did not exceed 300 μg.kg^−1^.

**Table 2:** Incidence and residual level (mean ± SD) of Drins and Chlordane in examined seafood species (μg.kg^−1^) of wet weight (*n* = 20 each).

		**Aldrin**	**Dieldrin**	**Endrin**	**∑DRINs**	***Oxy-*** **Chlordane**	***trans-*** **chlordane**	***trans-*** **nonachlor**	***cis-*** **Chlordane**	***cis-*** **nonachlor**	**∑CHLRs**
*C. harengus*	Incidence	20%	25%	ND		25%	20%	60%	25%	35%	
	Mean ± SD	3.75 ± 1.31	4.86 ± 1.33	ND	8.61 ± 2.64^a^	3.19 ± 1.92	4.40 ± 4.08	3.04 ± 2.77	3.55 ± 2.75	5.58 ± 4.39	19.75 ± 15.90^a^
*M. cephalus*	Incidence	85%	25%	ND		75%	20%	35%	35%	75%	
	Mean ± SD	1.61 ± 0.77	0.78 ± 0.04	ND	2.38 ± 0.81^b^	1.05 ± 0.80	0.84 ± 0.56	0.60 ± 0.39	0.42 ± 0.08	0.67 ± 0.26	3.59 ± 2.09^b^
*S. aurita*	Incidence	ND	ND	ND		20%	100%	ND	25%	35%	
	Mean ± SD	ND	ND	ND	ND	5.34 ± 5.18	11.45 ± 10.28	ND	1.88 ± 0.72	1.82 ± 1.38	20.48 ± 18.57^a^
*T. nilotica*	Incidence	50%	35%	ND		35%	85%	ND	100%	ND	
	Mean ± SD	0.88 ± 0.31	0.55 ± 0.02	ND	1.43 ± 0.33^b^	0.39 ± 0.02	0.63 ± 0.40	ND	0.49 ± 0.08	ND	1.51 ± 0.50^b^
*N. pelagicus*	Incidence	100%	ND	ND		ND	ND	100%	ND	ND	
	Mean ± SD	0.09 ± 0.01	ND	ND	0.09 ± 0.01^c^	ND	ND	0.03 ± 0.01	ND	ND	0.03 ± 0.01^c^
*S. savigngi*	Incidence	ND	ND	ND		40%	ND	40%	ND	25%	
	Mean ± SD	ND	ND	ND	ND	0.25 ± 0.01	ND	0.27 ± 0.07	ND	0.30 ± 0.02	0.82 ± 0.10^bc^

a, b, c Columns that carry different superscript letters are significantly different at P < 0.05. SAS 9.3 was used for all statistical analyses (SAS Institute Inc., Cary, NC, USA).

A popular brand of cyclodiene insecticides known as chlordane has been used extensively as termiticides and pesticides on residential properties and agricultural food crops (48). It can keep a strong bond with soil particles for up to 20 years and is extremely hydrophobic (49). The recorded data in Table 2, showed that the summation of chlordane residues (∑CHLRs) was 19.75 ± 15.90, 3.59 ± 2.09, 20.48 ± 18.57, 1.51 ± 0.50, 0.03 ± 0.01 and 0.82 ± 0.10 μg.kg^−1^ in examined *C. harengus, M. cephlaus, S. aurita, O. niloticus, N. pelagicus*, and *S. savigngi*, respectively. Chlordane residues were detected in fish samples from Cote d'Ivoire, Ghana, and Iran at concentrations 43.73, 3.44, and 0.083 μg.kg^−1^ (29, 32, 50). *S. savigngi* and *N. pelagicus* had much lower levels of chlordane residues than the other species under study (*P* < 0.05), which is likely due to their lower trophic status compared to fish (51). Regarding the acceptability of fish samples, all obtained chlordane values were lower than the permissible 300 μg.kg^−1^ according to the previous report (35).

Dichlorodiphenyltrichloroethane (DDT) was extensively used since its registration in 1946. DDT was primarily employed in agriculture to control pests that affected crop and livestock output, as well as in public health to combating the spread of the bubonic plague and malaria (52). According to reports from the WHO, DDT usage in global malaria prevention programs spared the deaths of more than five million people (53). The pp-DDE was dominant in all examined species within different concentrations of 5.08 ± 4.12, 0.98 ± 0.10, 3.07 ± 0.91, 0.93 ± 0.27, 0.08 ± 0.01 and 0.35 ± 0.02 μg.kg^−1^ in *C. harengus, M. cephlaus, S. aurita, O. niloticus, N. pelagicus*, and *S. savigngi*, respectively. Meanwhile, PP-DDT was detected only in three species 3.35 ± 2.84, 0.85 ± 0.05, and 6.53 ± 4.33 μg.kg^−1^ in *C. harengus, M. cephlaus*, and *S. aurita*, respectively (Table 3). The obtained results coincided with the authors (54) noted that epidemiological studies have used DDE as a biomarker for DDT exposure, also, agrees with the authors (55) who found pp-DDE in every fish sample from Ghana's Volta Lake. PP-DDE has also been found in fish muscle tissues from Lake Parishan in Iran (56).

**Table 3:** Incidence and mean residual level (Mean ± SD) of Dichlorodiphenyltrichloroethane (DDT) in examined seafood species (μg.kg^−1^) of wet weight (*n* = 20 each).

		**OP-DDE**	**PP-DDE**	**OP-DDD**	**PP-DDD**	**OP-DDT**	**PP-DDT**	**∑DDTs**
***C. harengus***	Incidence	ND	60%	20%	65%	ND	45%	
	Mean ± SD	ND	5.08 ± 4.12	9.21 ± 8.16	8.61 ± 2.64	ND	3.35 ± 2.84	18.25 ± 15.11^a^
***M. cephalus***	Incidence	25%	45%	35%	25%	50%	25%	
	Mean ± SD	0.79 ± 0.10	0.98 ± 0.10	0.76 ± 0.12	1.09 ± 0.29	0.83 ± 0.36	0.85 ± 0.05	5.30 ± 1.03^c^
***S. aurita***	Incidence	ND	20%	ND	35%	ND	20%	
	Mean ± SD	ND	3.07 ± 0.91	ND	2.98 ± 2.61	ND	6.53 ± 4.33	12.58 ± 7.58^b^
***T. nilotica***	Incidence	ND	100%	ND	85%	ND	ND	
	Mean ± SD	ND	0.93 ± 0.27	ND	0.55 ± 0.07	ND	ND	1.49 ± 0.33^d^
***N. pelagicus***	Incidence	ND	100%	ND	100%	ND	ND	
	Mean ± SD	ND	0.08 ± 0.01	ND	0.08 ± 0.02	ND	ND	0.16 ± 0.03^e^
***S. savigngi***	Incidence	ND	100%	ND	ND	ND	ND	
	Mean ± SD	ND	0.35 ± 0.02	ND	ND	ND	ND	0.35 ± 0.02^ed^

a, b, c, d, e Columns that carry different superscript letters are significantly different at P < 0.05. SAS 9.3 was used for all statistical analyses (SAS Institute Inc., Cary, NC, USA).

The recorded results in Table 3 revealed that ∑DDTs metabolites in descending form were 18.25 ± 15.11, 12.58 ± 7.58, 5.30 ± 1.03, 1.49 ± 0.33, 0.16 ± 0.03 and 0.35 ± 0.02 μg.kg^−1^ in examined *C. harengus, S. aurita, M. cephlaus, O. niloticus, N. pelagicus*, and *S. savigngi*, respectively. The ∑DDTs in fish samples from different countries were 140.63, 109.35, 0.8–82.9, 743, 8.97, and 6 in Kenya, Cote d'Ivoire, Turkey, Ethiopia and Nigeria (28, 29, 31, 45, 47, 57). The ∑DDTs in all examined species were below the MRL 1000 ppb (58).

**Figure 1:**
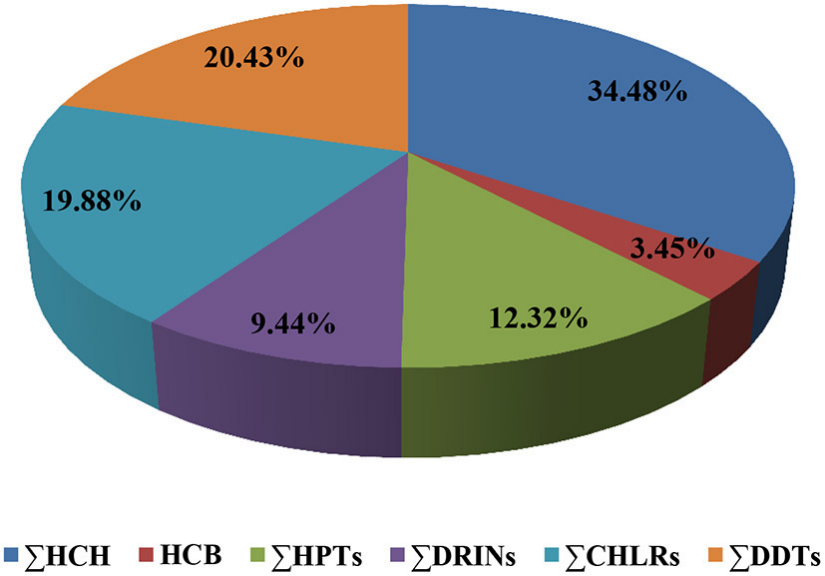
Distribution percentages of organochlorine pesticide groups in all examined seafood.

The discrepancies between the tested species could often be attributed to variations in environment and feeding strategies. It was also found that higher trophic species had a considerable increase in OCPs in their muscle compared to lower trophic species (59). Herein, in descending order, the contribution percentages of each OCP category collectively among species arranged as 34.485, 20.43 19.88, 12.32, 9.44, and 3.45 % for HCH, DDTs, CHLRs, HPTs, DRINs, and HCB, respectively (Figure 1). More information on the concentration percentages of the OCPs categories of each fish species is represented in Supplementary Figure 1. The obtained results in this study, like the results obtained previously (60) have revealed that *Stizostedion lucioperca* in Beysehir Lake has the highest pesticide residues overall for HCH derivatives. Another study in Turkey revealed that DDTs are the highest contaminant among OCPs (31).

### Risk assessment of detected organochlorine pesticide residues on human health

Fish consumption is thought to be a significant source of OCPs in humans. The potential for human exposure to OCPs was calculated using EDIs, the mean concentration of the target pollutants in fish. The data in Table 4 demonstrated that the EDIs of OCPs through eating various species of fish were significantly lower than the ADIs (61). The EDIs are low or on par with those in seafood (62, 63). The EDIs for HCHs in *N. pelagicus* and *S. savigngi* were nearly similar to seafood collected from Quanzhou and Xinghua Bay, Southeast China 0.00013 and 0.00024 (μg/kg/day), respectively. Also, herein EDIs of DDTs in *C. harengus* were comparable to the levels found in Nigeria, which were 0.012 and 0.013 (μg/kg/day), respectively (64). Meanwhile, the EDI of OCPs due to the consumption of fish collected from the Owan River in Nigeria exceeded the allowable daily average intake as a result of eating fish from the Owan River (65).

**Table 4:** Estimated daily intake (EDI μg/kg /day) of Organochlorine pesticides for Mansoura citizens, Egypt, due to seafood consumption in comparison to recommended acceptable daily intake (ADIs μg/kg /day).

**Organochlorine**	**∑HCHs**	**HCB**	**∑HPTs**	**∑Drins**	**∑CHLs**	**∑DDTs**
ADIs^a^	5	0.1	0.1	0.5	0.6	0.01
*C. harengus*	0.01367	0.00141	0.00707	0.00597	0.0137	0.01266
*M. cephalus*	0.00092	0.00065	0.00088	0.00165	0.00249	0.00368
*S. aurita*	0.04275	0.00109	0.00179	–	0.01421	0.00873
*T. nilotica*	0.00161	0.00024	0.00066	–	0.00105	0.00103
*N. pelagicus*	0.00019	–	0.00015	0.00006	0.00002	0.00011
*S. savigngi*	0.00099	0.00012	0.00022	–	0.00057	0.00024

ADIs, Acceptable daily intakes (μg/kg /day); EDIs, Estimated daily intakes (μg/kg /day),

a FAO/WHO (61).

**Figure 2:**
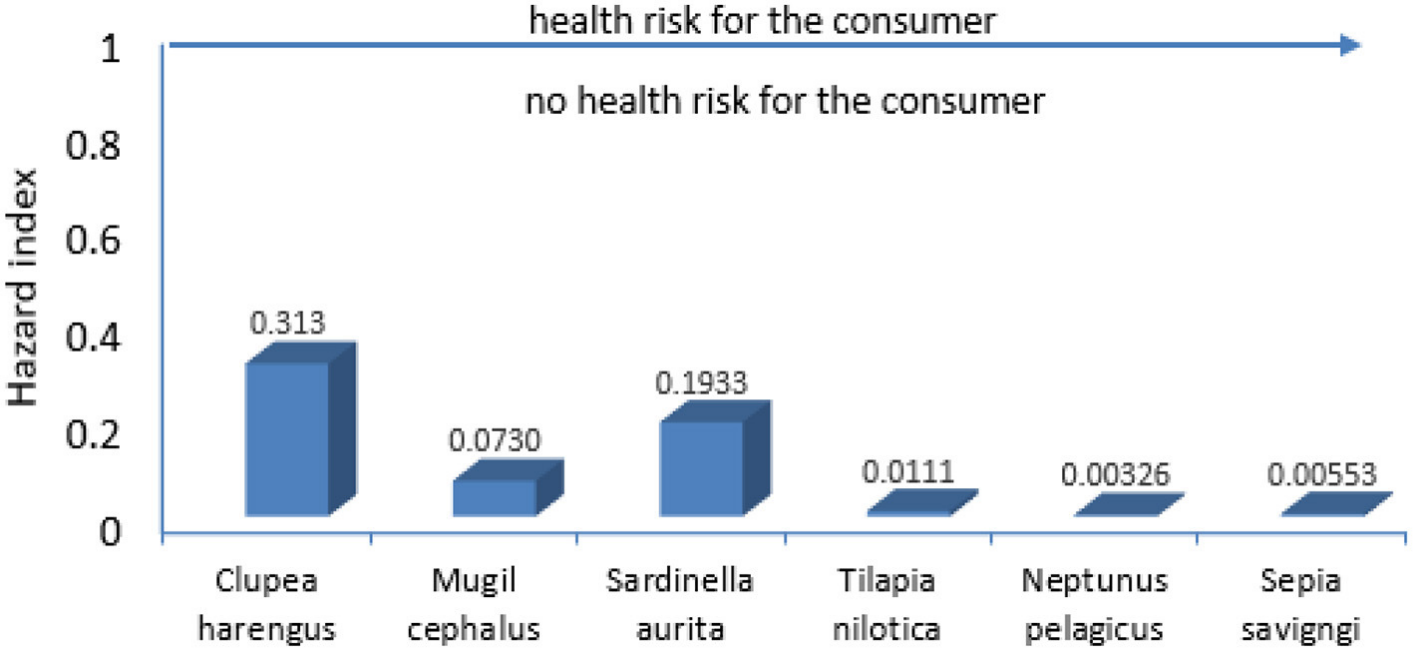
Hazard index for Mansoura citizens, Egypt, due to consumption of different seafood.

The non-carcinogenic indices of THQ and HI for OCPs in all examined species below or more than 1, are given in Table 5 and Figure 2, respectively, which indicate that consuming some fish species has no major damage to one's health. Nearly similar results from Wuhan, China, and South China revealed that THQ and HI were below 1 (66, 67). On contrary, THQ and HI exceeded 1 in many African countries, Nigeria's Lake Chad HI was 34.34 (45), Lagos Lagoon HI was 12.01 (30), and Lake Geriyo HI was 16.53 (68).

**Table 5:** Target hazard quotient (THQ) of organochlorine pesticides for Mansoura citizens, Egypt, due to seafood consumption.

**Organochlorine**	**∑HCHs**	**HCB**	**∑HPTs**	**∑Drins**	**∑CHLs**	**∑DDTs**
*C. harengus*	0.04556	0.00176	0.01414	0.19914	0.02741	0.02533
*M. cephalus*	0.00308	0.00082	0.00176	0.05505	0.00498	0.00735
*S. aurita*	0.1425	0.00136	0.00358	–	0.02842	0.01746
*T. nilotica*	0.00537	0.0003	0.00132	–	0.0021	0.00207
*N. pelagicus*	0.00062	–	0.00029	0.00208	0.00004	0.00022
*S. savigngi*	0.00331	0.00016	0.00044	–	0.00114	0.00049

## Conclusion

The level of OCPs in the edible tissue of seafood was lower than the regulatory level. Overall, the health risk associated with OCP exposure through seafood consumption was negligible. The findings demonstrated that organochlorines were under the FAO's permissible range.

## Data availability statement

The original contributions presented in the study are included in the article/Supplementary material, further inquiries can be directed to the corresponding authors.

## Ethics statement

The animal study was reviewed and approved by Institutional Review Board Statement: Animal Experimental Guidelines were followed, and the Animal Care and Use Committee of the Animal Health Research Institute, Zagazig University, and Egypt approved the experimental procedures.

## Author contributions

MH and WD jointly developed the hypothesis and concept of the study and contributed to the chemicals and materials preparations, as well as the techniques performed. For this research and scientific paper, OH, AT, IR, AS-A, FZ, and ZF are involved in the experimental procedures and analyses for this study and scientific paper. Each author contributed to the rewriting of the paper and the experimental analysis. All authors have read and approved the final manuscript.

## Funding

This study was supported by grant KEGA no. 009UVLF-4/2021: Innovation and implementation of new knowledge of scientific research and breeding practice to improve the teaching of foreign students on the subject of Animal Husbandry.

## Conflict of interest

The authors declare that the research was conducted in the absence of any commercial or financial relationships that could be construed as a potential conflict of interest.

## Publisher's note

All claims expressed in this article are solely those of the authors and do not necessarily represent those of their affiliated organizations, or those of the publisher, the editors and the reviewers. Any product that may be evaluated in this article, or claim that may be made by its manufacturer, is not guaranteed or endorsed by the publisher.

## Supplementary material

Supplementary Figure 1 The concentration of OCPs categories among six species of fish samples.
