# Supplementary material for: Health risk assessment of organochlorine pesticide residues in edible tissue of seafood
Source: Front Vet Sci. 2022 Nov 28;9:1042956. doi: 10.3389/fvets.2022.1042956 (PMC9761600; doi:10.3389/fvets.2022.1042956)
Supplement: Supplementary Figure 1 — The concentration of OCPs categories among six species of fish samples. [file Presentation_1.pptx]

## Slide 1
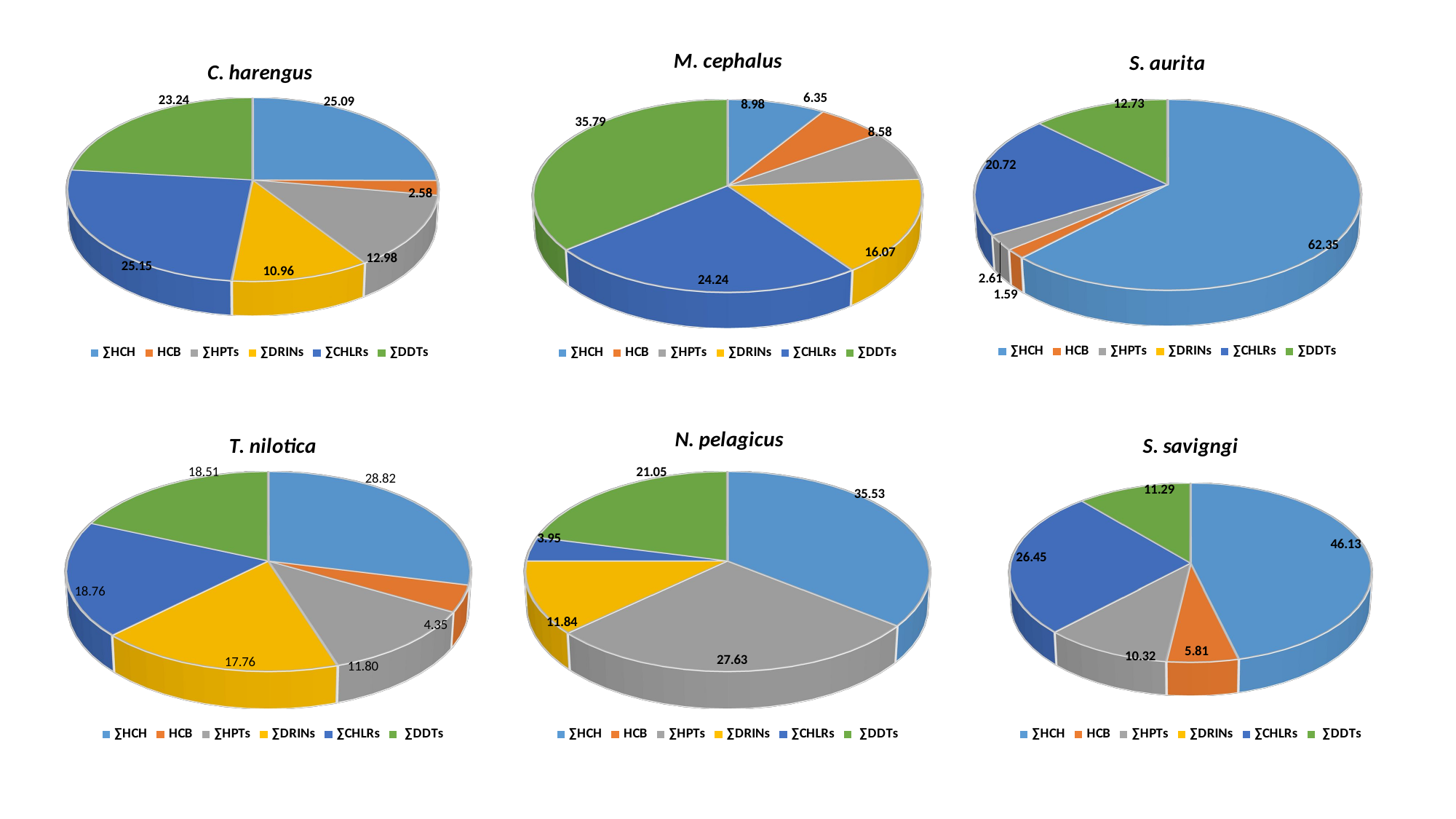

[unsupported chart]
[unsupported chart]
[unsupported chart]
[unsupported chart]
[unsupported chart]
[unsupported chart]

## Slide 2
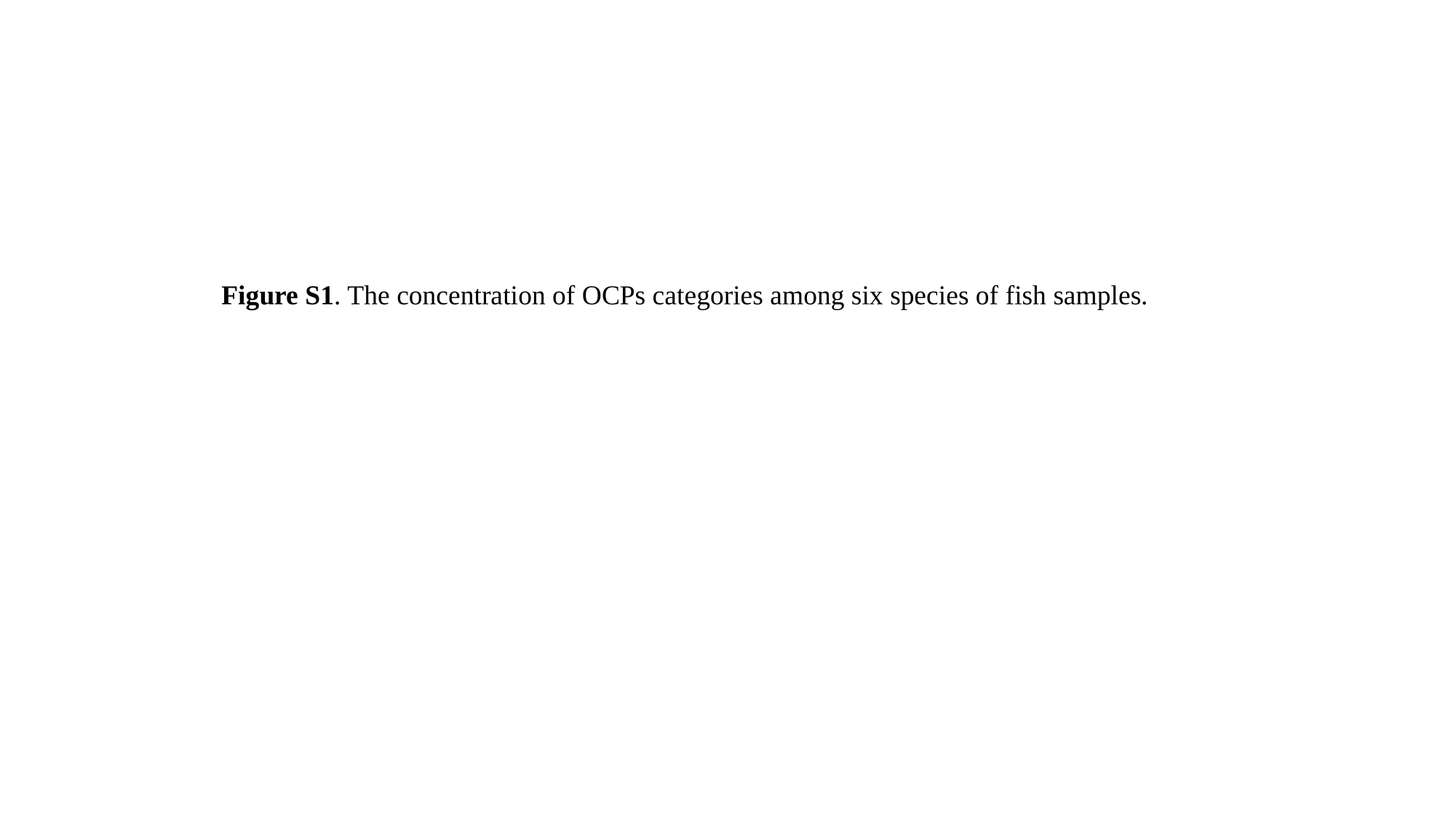

Figure S1. The concentration of OCPs categories among six species of fish samples.
